# Synergistic reproductive toxicity of lambda-cyhalothrin and thiamethoxam via oxidative stress and blood–testis barrier dysfunction in rats

**DOI:** 10.1038/s41598-026-59239-7

**Published:** 2026-07-13

**Authors:** Fatma M. El-Demerdash, Nahla M. Baraghit, Raghda A. El-Sayed, Hoda H. Baghdadi

**Affiliations:** Department of Environmental Studies, Institute of Graduate Studies and Research, Alexandria University, 163 Horreya Avenue, P.O. Box 832, Alexandria, 21526 Egypt

**Keywords:** Lambda-cyhalothrin, Thiamethoxam, Oxidative stress, Steroidogenic and antioxidant genes, BTB-related genes, Ultrastructural examination, Biochemistry, Molecular biology, Physiology, Zoology

## Abstract

Excessive pesticide use regularly pollutes the environment and endangers both human and animal health. The current study aimed to examine the main mechanism by which the combined action of pyrethroids, such as lambda-cyhalothrin (LC), and neonicotinoids, such as thiamethoxam, induced testicular toxicity and oxidative stress. Four groups of adult male rats were formed: control, Lambda-cyhalothrin (LC; 8 mg/kg B.W.), Thiamethoxam (TMX; 156 mg/kg B.W.), and LC + TMX groups. For 21 days, the rats received their doses orally. Results showed that LC and/or TMX caused significant alterations in body weight, gonadosomatic index, sperm quality, hormones, lipid peroxidation, DNA toxicity, and enzymatic and non-enzymatic antioxidants. Furthermore, testicular tissue showed biochemical, genes expression (steroid-forming acute regulatory protein (StAR), Cytochrome P450 17α-hydroxylase/17,20-lyase (CYP17a), luteinizing hormone receptor (LHR), cytochrome P450 cholesterol side-chain cleavage enzyme (P450scc), scavenger receptor class B type I (SR-B1), 3β-hydroxysteroid dehydrogenase (3β-HSD), superoxide dismutase (SOD), catalase (CAT) and glutathione peroxidase (GPx), Claudin-11, Occludin and ZO-1), and ultrastructural abnormalities. In conclusion, rats given both LC plus TMX had strong adverse effects, suggesting oxidative stress and reproductive toxicity.

## Introduction

Insecticide combinations are typically applied in agriculture to increase the protection against several pests attacking concurrently. Pest encounters pose a serious threat to crop yields intended for human consumption, with studies showing that exposure to pests reduces crop yields by more than half. Therefore, for several years, pest control has contributed to increased crop productivity globally^1,2^. A wide variety of pesticides, such as pyrethroids, organophosphates, and organochlorines, are accessible, and their use is increasing globally^3^. Pesticide use in modern agriculture has raised concerns about its hazardous impacts on non-target species, particularly humans^4^. The pesticides included in this study are classified as pyrethroids and neonicotinoids, two distinct families of insecticides. One of the most widely used insecticides worldwide is pyrethroids^5^. Pyrethroids, derived from *Chrysanthemum cinerariaefolium* flower, have been used since the 1970 s to manage insect pests in public health, agriculture, and livestock^6,7^. Lambda-cyhalothrin [a-cyano-3-phenoxybenzyl-3-(2-chloro-3,3,3-trifluoro-1-propenyl)−2,2- dimethylcyclo- propane carboxylate], a new generation type II synthetic pyrethroid, has a wide range of applications as an agropesticide, causes hypersensitivity, choreoathetosis, and salivation^8^. It is frequently applied to safeguard several food crops. Additionally, lambda-cyhalothrin is used in public health initiatives and veterinary treatments for farm animals to avoid and manage ectoparasites. Also, lambda-cyhalothrin remnants have been detected in fruits and vegetables, dairy cow milk, and blood, as well as in cattle meat^9^. Lambda-cyhalothrin exerts its insecticidal and toxic effects by disrupting the nervous system through prolonged opening of voltage-gated sodium channels, leading to hyperexcitation and paralysis, while also inducing oxidative stress and endocrine disruption in non-target organisms^10^.

Neonicotinoids have become increasingly popular because they specifically target nicotinic acetylcholine receptors (nAChRs) of insects and are thought to be less harmful to mammal species. Thiamethoxam (TMX), a common neonicotinoid of the second generation, is frequently used to control pests in different crops. However, growing research indicates that TMX may have significant toxicological consequences outside its intended targets, especially when it is converted into its metabolites that interact with nAChRs in mammals^11,12^. Despite its initial goal of lowering toxicity in vertebrates, research has revealed that TMX harms mammalian systems, resulting in oxidative stress, organ malfunction, and reproductive problems^13,14^. An increasing number of pesticides have been shown to negatively impact testicular function both in vivo and in vitro, raising concerns about testicular toxicity^15–18^. Furthermore, pesticides can induce oxidative stress, leading to biochemical and molecular disturbances and the generation of free radicals^17^. Studies on adult rats that explain how the pesticide mixture results in oxidative stress in tissues are currently limited. Thus, the purpose of the present investigation was to determine the effects of LC and TMX, each alone and in combination, on testicular toxicity, oxidative stress, biochemical, molecular, and tissue structural alterations in rats.

## Materials and methods

### Materials

Thiamethoxam: (EZ)−3-(2-chloro-1,3-thiazol-5-yl)methyl]−5-methyl-1,3,5-oxadiazinan-4-ylidene (nitro) amine (purity, 99.6%) was bought from Sigma-Aldrich (UK). Lambada λ-cyhalothrin technical grade (C_23_H_19_ClF_3_NO_3_), 97.8% purity (CAS number 91465-08- 6) was provided by Kafr El Zayat Pesticides and Chemicals Co. (Kafr El Zayat, Gharbia, Egypt).

### Experimental design

Twenty-eight male Wistar Albino rats (8–10 weeks of age, with an average body weight of about 160–170 g) were provided by the animal house of the Faculty of Medicine, Alexandria University, Alexandria, Egypt. The experimental plan was approved by the local Research Ethics Committee for Animal Care and Use at Alexandria University, Alexandria, Egypt (#AU14-240922-3-8), in accordance with guidelines of the National Institutes of Health for the care and use of laboratory animals. Rats were housed in stainless-steel bottomed wire cages under standard conditions (temperature of 22 ± 2 °C, relative humidity of 40–60%, and a 12-hour light/dark cycle). They were fed a pellet diet and had unlimited access to water. After two weeks of acclimation, rats were randomly assigned to four groups of seven. Group I: controls were given distilled water, group II: lambda-cyhalothrin (LC; 8 mg/Kg BW; 1/10 of the LD₅₀)^19^, group III: thiamethoxam (TMX; 156 mg/kg BW; 1/10 of the LD₅₀)^18^, and group IV: (LC + TMX). Animals received their doses orally by gavage for three weeks. After the end of the experimental period, rats were fasted overnight, euthanized using isoflurane, and then dissected, and their testes were removed.

### Preparation of serum samples and tissue homogenates

Samples of blood obtained by cardiac puncture were collected, put in glass tubes without anticoagulants, and left to stand at 25 °C for 30 min before being centrifuged for 15 min at 3000 xg. After extraction, sera were kept at −20 °C. In parallel, testis tissues were sliced and homogenized (10% w/v) in cold 10 mM phosphate buffer (pH 7.4). The homogenate was centrifuged at 10,000 xg for 20 min at 4 °C, and the supernatants were saved for use in subsequent assays.

### Evaluation of sperm quality

To allow the spermatozoa to diffuse into the fluid, the left caudal portion of each testicle’s epididymis was carefully taken off, diced in 5 mL of Hanks’ buffered salt solution, and allowed to stand for 15 min at room temperature. A bright-field microscope (Olympus, Tokyo, Japan) and computer-assisted semen analysis were used to assess the sperm features^20^. Using the Blazak et al.^21^, approach, the daily generation of sperm in the rat testis was measured.

### Reproductive hormones analysis

An enzyme-linked immunosorbent assay (ELISA) kit (Cusabio, USA, Catalog number: CSBE05097r) was used to measure testosterone (T) level in accordance with the manufacturer’s instructions. To quantify luteinizing hormone (LH) level, an RIA kit from NIADDK, Bethesda, MD, USA, was used. An ELISA kit (DiaMetra, Via Giustozzi, Italy) and immunodiagnostic reagents were used to assess follicle-stimulating hormone (FSH) in rat serum.

### Assessment of oxidative stress and antioxidant biomarkers

Thiobarbituric acid-reactive substances (TBARS), hydrogen peroxide (H_2_O_2_) levels, and reduced glutathione (GSH) content were determined in testis homogenate using (TBARS assay kit, EEA021) from Thermo Fisher Scientific, reduced glutathione (G4251) from Sigma-Aldrich, and hydrogen peroxide (386790) from Sigma-Aldrich. Additionally, superoxide dismutase (SOD; EC 1.15.1.1), catalase (CAT; EC 1.11.1.6), glutathione reductase (GR; EC 1.8.1.7), glutathione peroxidase (GPx, EC 1.11.1.9), and glutathione S-transferase (GST; EC 2.5.1.18) activities were measured using commercially available kits (Biodiagnostic, Egypt).

### Evaluation of DNA toxicity

The 8-hydroxy-2′-deoxyguanosine (8-OH-2DG) technique was used to assess DNA damage in serum. The phenol-chloroform procedure was used to extract DNA from serum. An ELISA-based kit (Catalogue No. ab201734, Supplier: Abcam) was used to quantify 8-OH-2DG levels.

### Quantitative real-time PCR

SYBR Green PCR Master Mix (2x SensiFast SYBR, Bioline, catlog No. Bio-98 002), real-time PCR was used to measure the relative amounts of the mRNA levels of steroid-forming acute regulatory protein (StAR), Cytochrome P450 17α-hydroxylase/17,20-lyase (CYP17a), luteinizing hormone receptor (LHR), cytochrome P450 cholesterol side-chain cleavage enzyme (P450scc), scavenger receptor class B type I (SR-B1), 3β-hydroxysteroid dehydrogenase (3β-HSD), superoxide dismutase (SOD), catalase (CAT) and glutathione peroxidase (GPx), Claudin-11, Occludin and ZO-1 in rat testes samples. Table 1 displays these primer sequences. As an internal control, the housekeeping gene β-actin was employed. The expression of β-actin was used to determine relative gene expression in each treatment group relative to the control using the 2^−ΔΔCt^ approach, as previously described by Pfaffl^22^.

### Ultrastructural examination

Fresh testis tissue sections (less than 1 mm^3^) were immediately fixed by submerging them in “4% formaldehyde and 1% glutaraldehyde (4F:1G)” and phosphate buffer (pH 7.2) at 4 °C for three hours. After that, they were fixed with 1% citric acid, cleaned with 0.1 M phosphate-buffered saline (PBS), and then rinsed once more with PBS. Before applying dye with uranyl acetate and lead citrate, the segments underwent the following processes: dehydration, soaking, embedding, polymerization, chopping, and slicing. A transmission electron microscope (TEM) (Hitachi H-7650, Tokyo, Japan) was used to examine testis ultrastructure sections. The identification of testicular cell types (spermatogonia, Sertoli cells, primary spermatocytes) in TEM micrographs was performed based on established ultrastructural morphological criteria, including their characteristic nuclear shape, chromatin pattern, nucleolar appearance, and distinct location within the seminiferous epithelium. Specifically, ultrastructural changes (e.g., mitochondrial degeneration, nuclear irregularities, vacuolization, disruption of Sertoli–germ cell junctions, and basement membrane alterations) were evaluated using a scoring system based on the severity and frequency of lesions observed across multiple fields. The degree of damage was graded as follows: - (normal), + (mild alterations), ++ (moderate damage), and +++ (severe damage). The scoring was performed on randomly selected micrographs from each group in a blinded manner.

### Statistical analysis

The results of each experiment were displayed as the mean ± standard error of mean (SEM), with *P* < 0.05 designated as the significance level. Means were compared using Graph Pad Prism 5 software (San Diego, CA, USA) and the post hoc test Tukey’s Honestly Significant Difference (Tukey’s HSD) and one-way Analysis of Variance (ANOVA).

## Results

No mortality was observed in rats treated with LC and/or TMX, and no clinical signs of toxicity, such as loss of fur, salivation, urination, or lethargic activity.

### Body and organ weights

Body weights, absolute reproductive organ weights, and relative reproductive organ weights of control and experimental male rats are presented in Table 2. The final body weights of the LTC and/or TMX-treated rats were significantly (*P* < 0.05) decreased when compared with the control animals.

### Sperm analysis

Rats treated with LC and/or TMX showed a significant elevation in serum 8-hydroxy-2′-deoxyguanosine (8-OH-2DG) levels and sperm abnormality and a decrease in the levels of serum T, LH, and FSH and testicular daily sperm production (DSP), sperm count, and motility as compared to the control group after 3 weeks of administration (*P* < 0.05). The observed alterations in the LC plus TMX group were statistically significant (*P* < 0.05) compared to the single-treated groups (Table 3).

### Oxidant stress and antioxidant biomarkers

**Fig. 1 Letters above the columns are not found. Please replace this figure with the attached file:**
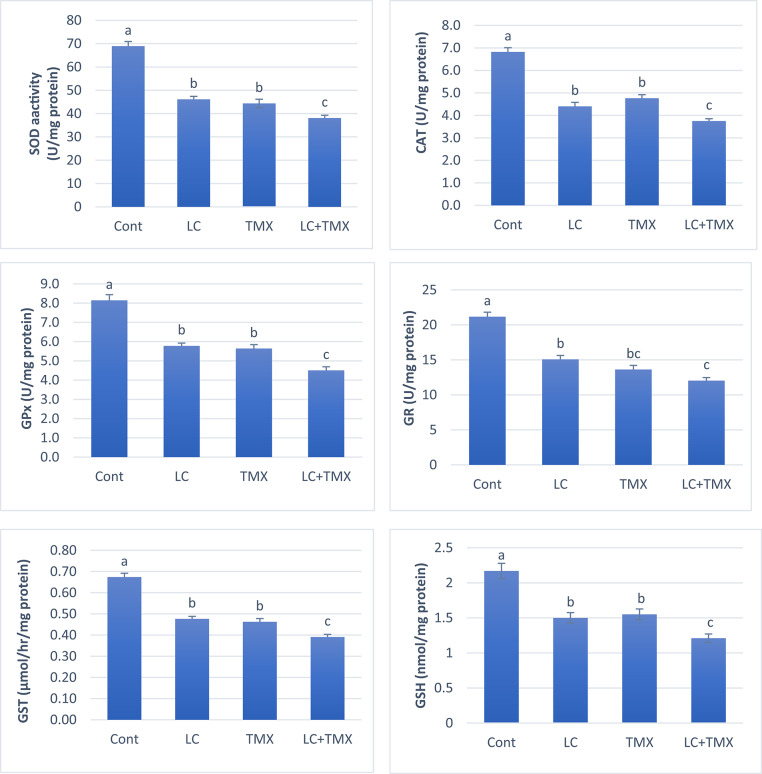
Effect of LC, TMX, and LC + TMX on testicular enzymatic and non-enzymatic antioxidants in rats. Values for each treatment group are given as means ± SE; *n* = 7/group. The mean values of LC, TMX, and LC + TMX that did not share common letters (a, b, and c) were substantially different at *P* < 0.05 compared to the control group. Group comparison: LC, TMX, and LC + TMX are contrasted with the control group.

Results indicated that TBARS and H_2_O_2_ concentrations were significantly (*P* < 0.05) increased; however, GSH content and SOD, CAT, GPX, GR, and GST activities were significantly decreased in the testes homogenate of rats treated with LC and/or TMX as compared to the control. Rats treated with both TMX and LC showed a higher increase in TBARS and H_2_O_2_ levels, and a decrease in GSH, SOD, CAT, GPX, GR, and GST as compared to the control group (Table 4; Fig. 1).

### Expression of testicular steroidogenic, antioxidant, and blood–testis barrier genes

**Fig. 2:**
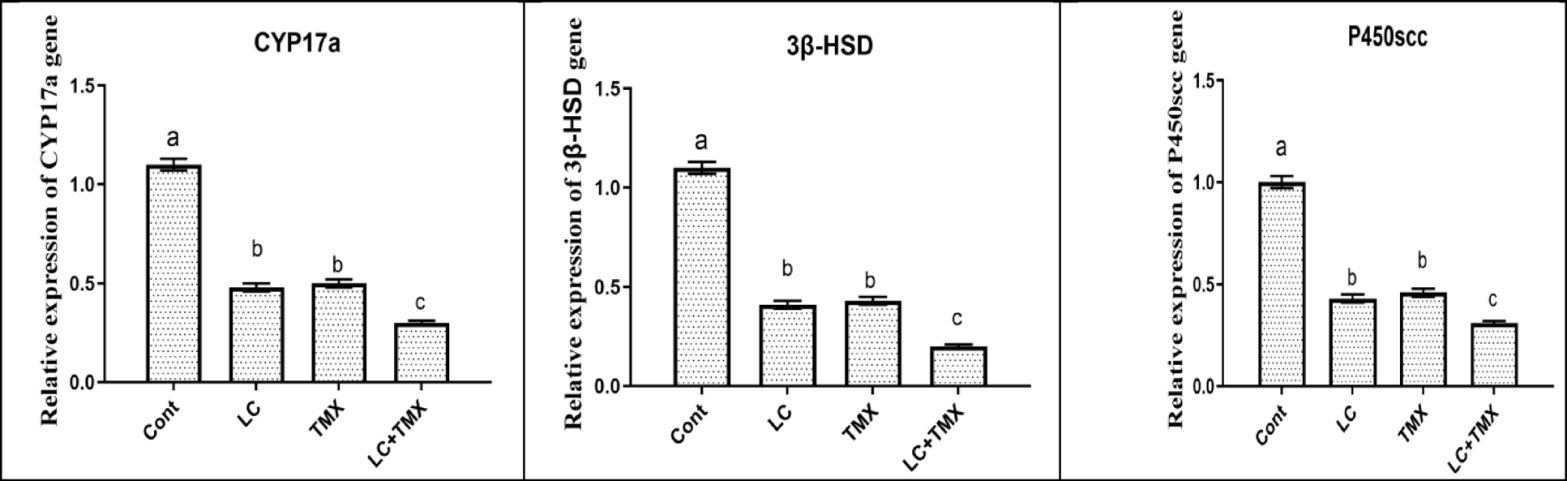
Enzymatic relative gene expressions in rat testes after exposure to LC and/or TMX. Values for each treatment group are given as means ± SD; *n* = 3/group. The mean values of LC, TMX, and LC + TMX that did not share common letters (a, b, and c) were substantially different at *P* < 0.05 compared to the control group. Group comparison: LC, TMX, and LC + TMX are contrasted with the control group.

**Fig. 3:**
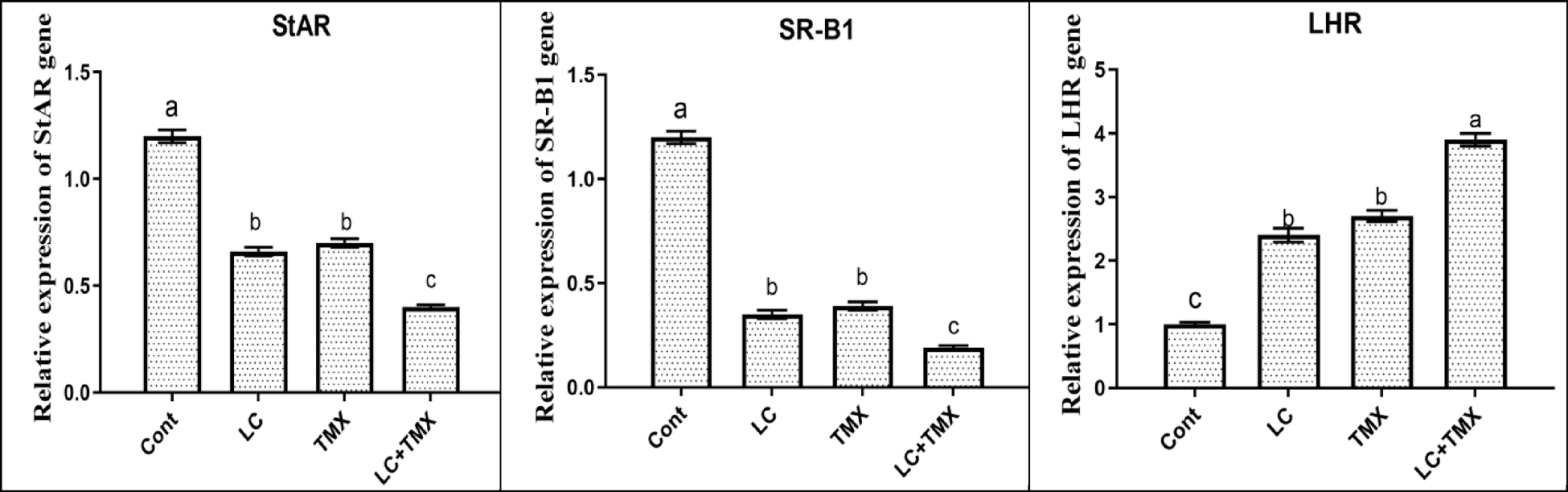
Cholesterol uptake-related genes and hormone-related gene expressions in testes of rats treated with LC and/or TMX. Values for each treatment group are given as means ± SD; *n* = 3/group. The mean values of LC, TMX, and LC + TMX that did not share common letters (a, b, and c) were substantially different at *P* < 0.05 compared to the control group. Group comparison: LC, TMX, and LC + TMX are contrasted with the control group.

**Fig. 4:**
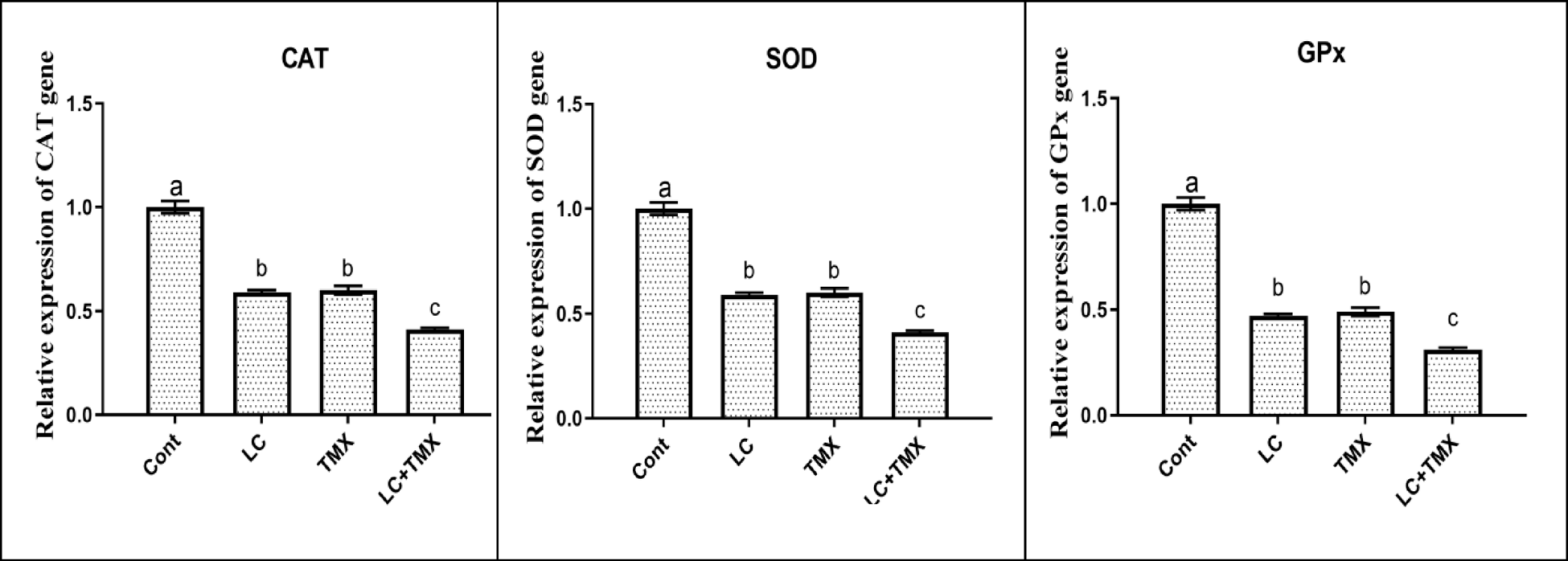
Antioxidant relative gene expressions in the testes of rats treated with LC and/or TMX. Values for each treatment group are given as means ± SD; *n* = 3/group. The mean values of LC, TMX, and LC + TMX that did not share common letters (a, b, and c) were substantially different at *P* < 0.05 compared to the control group. Group comparison: LC, TMX, and LC + TMX are contrasted with the control group.

**Fig. 5:**
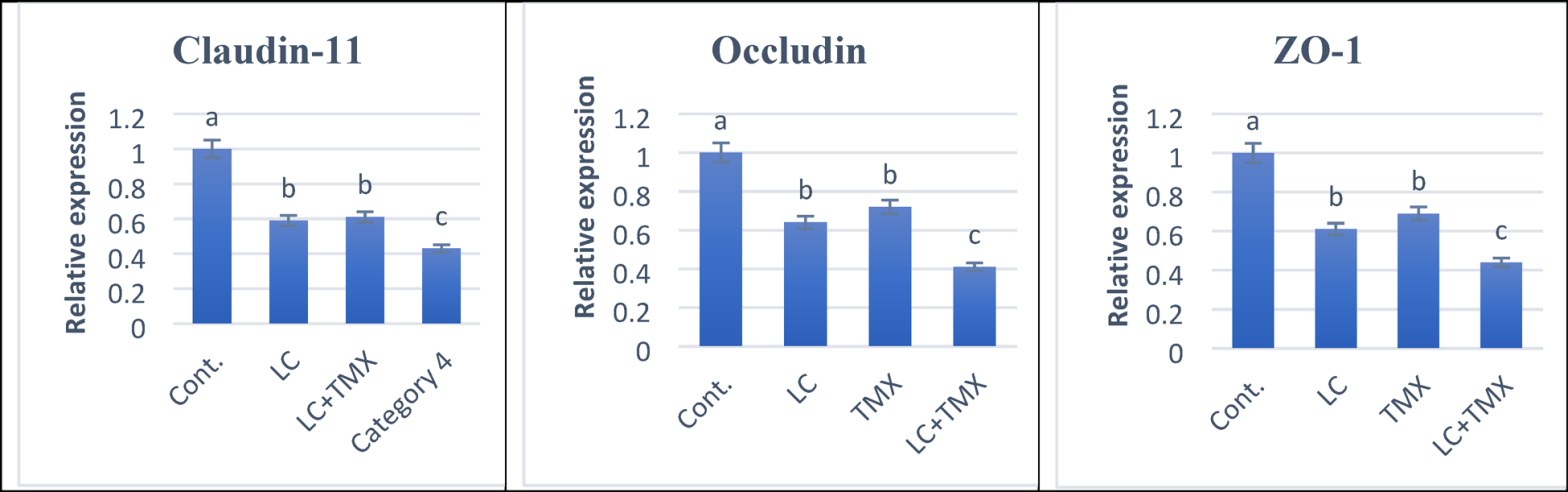
Blood–testis barrier–related gene expressions in the testes of rats treated with LC and/or TMX. Values for each treatment group are given as means ± SD; *n* = 3/group. The mean values of LC, TMX, and LC + TMX that did not share common letters (a, b, and c) were substantially different at *P* < 0.05 compared to the control group. Group comparison: LC, TMX, and LC + TMX are contrasted with the control group.

Notably, the mRNA expression of selected testicular steroidogenic, antioxidant and blood–testis barrier–related genes was altered in testicular tissues of LC and/or TMX-exposed rats. Compared with the control group, StAR, CYP17a, 3β-HSD, SR-B1, P450scc, CAT, SOD, Gpx, Claudin-11, Occludin, and ZO-1 genes were significantly (*P* < 0.05) downregulated in the LC and/or TMX group, whereas the LHR gene was significantly (*P* < 0.05) upregulated (Figs. 2, 3, 4, 5).

### Testis ultrastructure

**Fig. 6:**
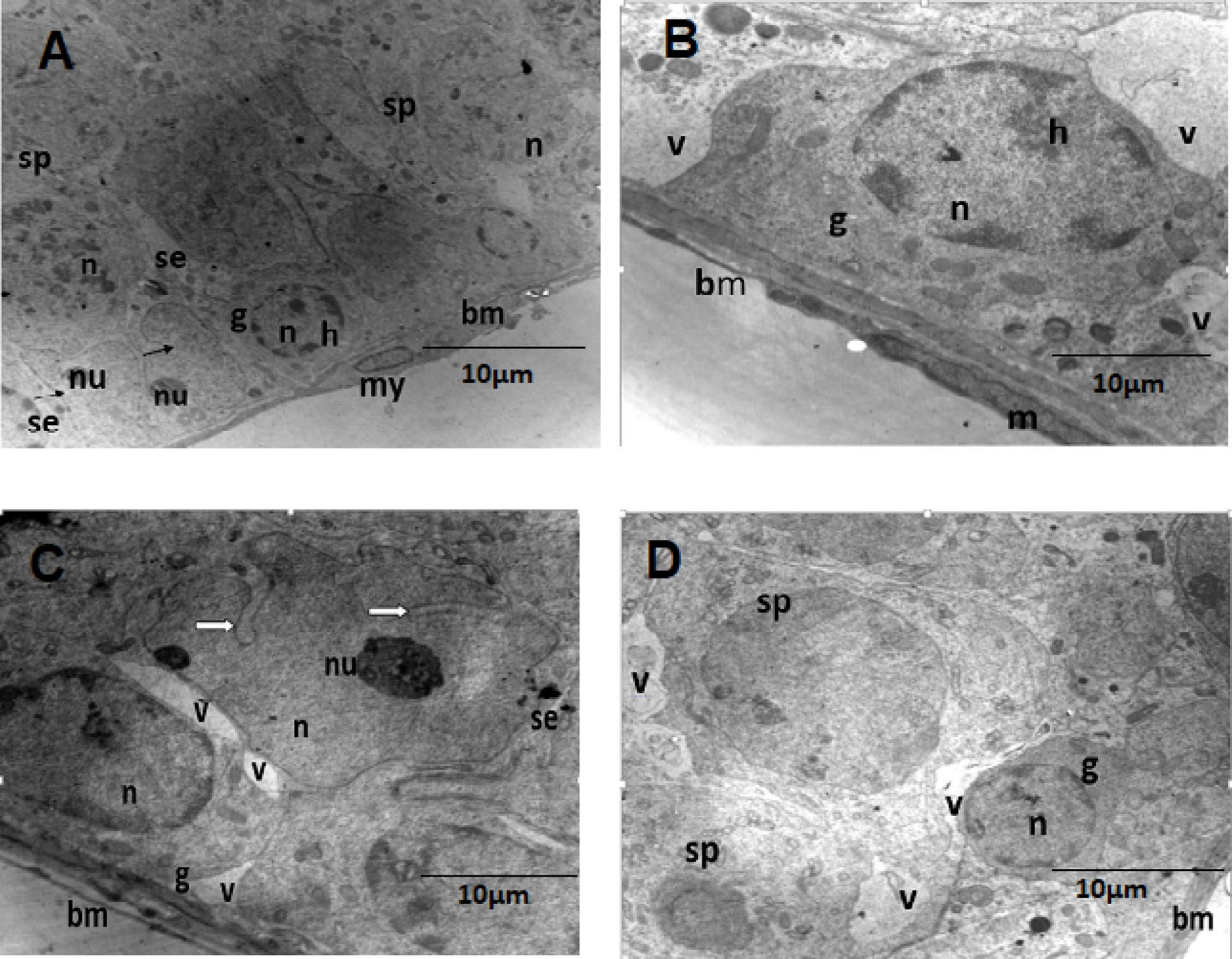
(**A**-**D**) A transmission electron micrograph of a control (**A**) rat testis showing spermatogonia (g) with a round nucleus (n) and coarse clumps of marginated heterochromatin (h). Two adjacent Sertoli cells (se) with nuclear pockets (arrow) and a prominent nucleolus (nu) join together by a light junction forming the blood–testis barrier. Primary spermatocyte (sp) with large, rounded nuclei (n) (TEM, X1000). Lambda cyhalothrin (**B**), revealing spermatogonia (g) with a round nucleus (n) and marginated heterochromatin (h) resting on the basement membrane (bm) and myoid cell (my) outside the basement membrane (TEM, X3000). Thiamesoxam (**C**) illustrating spermatogonia (g) with oval nuclei (n) resting on the basement membrane (bm). Also, Sertoli cell (se) with irregular nuclear membrane (arrows) and prominent nucleolus (nu). Vacuolization (v) appears between spermatogonia and Sertoli cells (TEM, X2000). LC and TMX (**D**) showing two abnormal primary spermatocytes (sp). Also, abnormal position of spermatogonia (g) with rounded nuclei (n). Vacuoles were also seen (v) (TEM, X1500). Cell types were identified using widely accepted ultrastructural criteria. Spermatogonia (g) are situated on the basement membrane, characterized by a round/oval nucleus and marginal heterochromatin. Sertoli cells (se) exhibit an irregular nucleus with a prominent nucleolus. Primary spermatocytes (sp) are characterized by their large, rounded nuclei within the abluminal compartment.

The ultrastructural examination of control testes of rats revealed that the seminiferous epithelium consists of two cell populations, non-dividing somatic Sertoli cells and highly proliferating germ cells. An electron micrograph of rat testis treated with LC and/or TMX showed damage in testicular constituents, especially in the combination group (Fig. 6A- D) and (Table 5).

## Discussion

The widespread use of pesticides without proper control leads to serious environmental contamination and health risks. It disrupts the male reproductive system and potentially affects male fertility^23^. Pyrethroids and neonicotinoid insecticides are the most often employed in agriculture nowadays. The lack of knowledge about how LC + TMX affects the rat testes makes it extremely challenging to compare our findings with those of other authors. In the present investigation, hypophagia or weight loss induced by the LC’s direct cytotoxic action may be the cause of final body weight reduction. In agreement, animals treated with LC displayed decreased body weight, feed consumption, and relative testicular and epididymal weights^9,24^. Additionally, the weight growth of the rats exposed to TMX was significantly decreased. This may be due to the TMX’s quick effects on the digestive tract and poor nutritional absorption^25,26^.

As pro-oxidants, pesticides affect several organs^27^. Human beings are at risk from synthetic pyrethroid pesticides, particularly those who work in their manufacturing, use them in agriculture, and eat contaminated food. The biological membrane is the main site of pyrethroid action due to their great hydrophobicity^28^. LC, a type II pyrethroid with an α-cyano moiety, may be hazardous because it produces cyanohydrins, which are unsettled under physiological circumstances and may eventually break down into cyanides and aldehydes, potentially contributing to the production of free radicals^29^. The increased TBARS and H_2_O_2_ levels in rats intoxicated with LC and/or TMX are consistent with earlier research suggesting that pyrethroid-induced oxidative damage may be due to their lipophilicity, which enables them to cross cell membranes^30,31^. Also, neonicotinoids cause oxidative damage and ROS generation in a variety of organs^32^.

The ratio of oxidant generation to antioxidant elimination determines the level of LPO^33^. Glutathione is a significant non-enzymatic antioxidant^15^. The observed decrease in GSH concentration may be due to free radicals produced by insecticides^34,35^. The counterbalancing action of antioxidant enzymes is lost when insecticides generate excessive ROS^36,37^. The decline in examined antioxidant enzymes is consistent with findings of El-Demerdash^38^ and Saraivaa et al.^39^. GST is an essential endogenous detoxifying enzyme^19,40,41^. Furthermore, GPx aids in the reduction of H_2_O_2_^42,43^. Interestingly, superoxide radicals are converted to H_2_O_2_ by SOD, whereas H_2_O_2_ is transferred to water by CAT^44^. Thus, antioxidant enzymes can reduce ROS-damaging effects and actively protect against oxidative cellular damage.

The present study provides compelling evidence that subacute exposure to Lambda-cyhalothrin (LC) and Thiamethoxam (TMX) induces significant reproductive toxicity in male rats by oxidative stress, endocrine disruption, and direct damage to spermatogenesis. The most critical finding is the significant elevation in serum 8-hydroxy-2′-deoxyguanosine (8-OH-dG) in all treated groups^45^. The consequences of this are profound: oxidative DNA damage in germ cells can induce mutations and trigger apoptosis^46^. The observed decline in testicular daily sperm production (DSP), sperm count, and motility is a direct functional consequence of this oxidative damage. The seminiferous epithelium is highly vulnerable to oxidative stress^47^. The strong correlation between high 8-OH-dG levels and poor sperm parameters underscores oxidative stress as a central pillar of the observed testicular pathology.

Crucially, our results pinpoint a specific disruption\ of the hypothalamic-pituitary-gonadal (HPG) axis as a key mechanism. The significant reduction in circulating FSH, LH, and T levels reveals a cascade of endocrine failure. The suppression of these pituitary gonadotropins suggests that LC and TMX may exert their toxicity at a central level^48^. Furthermore, pyrethroids and neonicotinoids have been shown to directly inhibit key steroidogenic enzymes^49^. The dramatic fall in testosterone levels directly explains the atrophy of the androgen-dependent seminal vesicles and ventral prostate. TMX’s endocrine-disrupting properties are demonstrated by its impact on reproductive health, including lower testosterone levels, sperm characteristics, and organ weights. These results corroborate recent claims that TMX metabolites can disrupt spermatogenesis and hormone control^50^. Lambda-cyhalothrin exposure in male rats significantly impairs sperm characteristics and affects hormonal balance^51,52^.

Exposure to LC and/or TMX significantly reduced serum testosterone and downregulated key steroidogenic genes (StAR, CYP17a, 3β-HSD, SR-B1, P450scc), consistent with the effects of other neonicotinoids^48,53,54^. As testosterone is vital for reproduction, its impairment causes dysfunction^53^. The mechanism likely involves direct damage to Leydig cells, disrupting the steroidogenic pathway^54,55^. The concurrent alteration in LHR expression suggests a disruption in the LH signaling cascade^32,56^. This is supported by previous findings that TMX inhibits steroidogenic enzyme activity and StAR expression^57,58^.

The current investigation showed that exposure to LC and/or TMX significantly reduced the expression of Claudin-11, Occludin, and ZO-1 mRNA in rat testicular tissue. These proteins are crucial components of the blood–testis barrier (BTB)^59,60^. Claudin-11 is regarded as the primary structural claudin isoform of the BTB. Claudin-11 has an essential function in testicular physiology^61,62^. Thus, the present study’s observation of Claudin-11 transcriptional suppression strongly implies that the BTB is structurally compromised. Disruption of Sertoli cell junctional integrity is further supported by reduced expression of ZO-1 and Occludin. The downregulation or redistribution of these proteins has frequently been used as molecular evidence of BTB malfunction^60^. It is well known that cytoskeletal disarray induced by oxidative stress can disrupt ZO-1 anchoring and alter occludin expression^63^.

Inflammatory signaling and oxidative stress are probably the causes behind the observed gene suppression. In rat tissues, exposure to lambda-cyhalothrin has been shown to induce lipid peroxidation, increase ROS, and weaken antioxidant defenses^9^. Stress-responsive kinases and transcription factors that adversely control tight junction gene expression can be activated by excess ROS^63^. Similarly, exposure to thiamethoxam has been linked to reproductive toxicity and systemic oxidative stress in male rats^64^. BTB dysfunction may also be caused by endocrine disturbance. According to Mruk and Cheng^60^, testosterone tightly regulates the dynamics of Sertoli cell junctions. According to reports, pyrethroids and neonicotinoids disrupt reproductive hormone balance and endocrine signaling^9,64,65^.

The observed reduction in testosterone levels is likely attributable to oxidative stress–induced Leydig cell dysfunction, possible DNA damage in testicular cells, downregulation of key steroidogenic enzymes, and disruption of the hypothalamic–pituitary–gonadal axis, rather than a direct consequence of DNA damage alone^66^. Rats treated with LC showed partial to total breakdown of Leydig cells in the interstitial space. High levels of ROS cause oxidative stress, which damages the testicular tissues when LC is deposited there^56^. Low blood testosterone levels are caused by LC’s direct action on the testes^67^. In rats given cypermethrin, spermatogenesis is suppressed by low serum testosterone^67,68^. In agreement, exposure to LC significantly reduced germinal, Leydig, and spermatocyte cells^69,70^. Also, cyhalothrin therapy caused pathological damage to the architecture of the seminiferous tubules. So, increased ROS levels may reduce the effective concentration of antioxidants^71^. Testes of TMX-treated rats displayed degeneration of the Leydig cells, spermatogonia, and spermatocytes. Despite their decreased affinity for mammalian nAChRs, neonicotinoid insecticides have been shown to reduce mammalian reproduction^72^. TMX exposure negatively impacted reproductive characteristics such as T levels, sperm quality, and histopathological abnormalities^73^. The importance of TMX in producing oxidative DNA damage in rat testes was also emphasized by Abdel-Razik et al.^13^. The effects in the combination group were consistently more severe than in either single-treated group. This is a significant public health concern, as co-exposure to multiple pesticide classes is the norm rather than the exception^74^.

The current findings support a multilayered process in which oxidative stress causes testicular failure. Excess ROS (TBARS and H₂O₂) and depleted antioxidants alter redox-sensitive pathways and mitochondrial activity in Leydig cells. This lowers StAR and steroidogenic enzymes (CYP17a, 3β-HSD, P450scc), resulting in low testosterone production. Concurrently, oxidative DNA damage (increased 8-OHdG) undermines genomic integrity in germ and somatic cells, impeding spermatogenesis. ROS also affects the blood–testis barrier by reorganizing the cytoskeleton and deregulating junctional proteins (Claudin-11, Occludin, and ZO-1)^63^. Local effects are exacerbated by systemic endocrine disturbance; low LH and FSH levels indicate hypothalamic-pituitary-gonadal axis dysfunction. LC and TMX co-exposure produce reproductive damage via an integrated network of oxidative stress, mitochondrial malfunction, gene dysregulation, and hormonal imbalance, rather than a single mechanism^48^.

## Conclusion

Conclusively, LC and/or TMX intoxication in rats led to oxidative stress, perturbations in the antioxidant defense status, biochemical markers, and expression of genes associated with steroidogenic and antioxidant functions. Furthermore, changes in sperm quality, hormone levels, and DNA damage are noted. Additionally, ultrastructural changes in testicular tissue were detected. Moreover, exposure to thiamethoxam and lambda-cyhalothrin impairs the transcription of important BTB-associated genes in rat testicular tissue. The more pronounced effect in the combination group suggests a synergistic or additive generation of ROS, pointing to a shared pathway of oxidative insult that is exacerbated when the pesticides are co-administered. This synergy arises from complementary mechanisms, where dual excitotoxicity overwhelms antioxidant defenses, causing cellular apoptosis and inflammation. Also, to prove functional barrier disruption and identify the specific molecular processes implicated, future research combining protein expression analyses, permeability experiments, and hormone profiling is necessary. Future work must focus on elucidating the precise mechanistic interplay and expanding into long-term, low-dose, and multi-generational studies.

**Table 1:** Primers used for real-time PCR amplifications.

Gene	GenBank accession number	Oligonucleotide sequence
StAR	NM_031558	f5^/^-GGGCATACTCAACAACCAG-3^/^
		r5^/^-ACCTCCAGTCGGAACACC-3^/^
CYP17a	NM_012753	f5^/^-CTCTGGGCACTGCATCAC-3^/^
		r5^/^-CAAGTAACTCTGCGTGGGT-3^/^
LHR	NM_012978	f5^/^-CATTCAATGGGACGACTCTA-3^/^
		r5^/^-GCCTGCAATTTGGTGGA-3^/^
3β-HSD	M38178	f5^/^-TGTGCCAGCCTTCATCTAC-3^/^
		r5^/^-CTTCTCGGCCATCCTTTT-3^/^
SR-B1	AY451993	f5^/^-ACAGGTCCCAGGGCTCAG-3^/^
		r5^/^-CGTGCGGTTCATAAAGG-3^/^
P450SCC	J05156	f5^/^-CTTTGGTGCAGGTGGCTAG-3/
		r5^/^-CGGAAGTGCGTGGTGTTT-3^/^
CAT	NM_012520.2	f5^/^- GGAGAGGCAGTGTACTGCAA-3^/^
		r5^/^- TTGCCACTGGCGATGGCATT-3^/^
SOD	X05634.1	f5^/^- GAAGGCCGTGTGCGTGCTGA – 3^/^
		f5^/^- CCTTCAGTTAATCCTGTAATC-3^/^
Gpx	NM_030826.4	f5^/^- CCGTGTATGCCTTCTCCGCG-3^/^
		r5^/^- TGCCTCAGAGGGACGCGACA – 3^/^
Claudin-11	NM_053457	f5′-TGGGAGGATGGAAGTTTGGG-3′
		r5′-CAGAGGAGACACGAGGAGGA-3′
Occludin	NM_031329	f5′-TGGAGTGGCAGGTTGTGTTT-3′
		r5′-CAGCTGCTGTTGCTGTTGTT-3′
ZO-1	NM_009386	f5′-AGCAGGAAACAGGAGGACGA-3′
		r5′-GCTGGAAGAGGAGGAGGTGA-3′
β-actin	NM_031144	f5^/^-TCGTGCGTGACATTAAAGAG-3^/^
		r5^/^-ATTGCCGATAGTGATGACCT-3^/^

**Table 2:** Body weight and reproductive organ weights in rats intoxicated with LC, TMX, and LC + TMX.

Parameters	Control	LC	TMX	LC + TMX
Initial body weights (g)	169.56 ± 4.42	168.80 ± 3.25	169.32 ± 2.11	168.02 ± 2.02
Final body weights (g)	234.12 ± 6.3^a^	204.43 ± 5.75^b^	209.58 ± 6.19^b^	198.03 ± 4.75^c^
Absolute testes weight (g)	2.12 ± 0.03^a^	1.63 ± 0.02^b^	1.71 ± 0.01^b^	1.32 ± 0.01^c^
Relative testes weight (g/100 g body weight)	0.905 ± 0.02^a^	0.797 ± 0.02^b^	0.815 ± 0.04^b^	0.666 ± 0.07^c^
Absolute epididymis weight (g)	0.34 ± 0.07^a^	0.24 ± 0.03^b^	0.27 ± 0.06^b^	0.19 ± 0.01^c^
Relative epididymis weight (g/100 g body weight)	0.145 ± 0.03^a^	0.117 ± 0.01^b^	0.128 ± 0.05^b^	0.095 ± 0.01^c^
Absolute seminal vesicle weight (g)	0.89 ± 0.12^a^	0.75 ± 0.03^b^	0.77 ± 0.19^b^	0.51 ± 0.02^c^
Relative seminal vesicle weight (g/100 g body weight)	0.389 ± 0.09^a^	0.366 ± 0.04^b^	0.367 ± 0.19^b^	0.257 ± 0.03^c^

Values for each treatment group are given as means ± SE; *n* = 7/group. The mean values of LC, TMX, and LC + TMX within a row that did not share common superscript letters (a, b, and c) were substantially different at *P* < 0.05 compared to the control group.

Relative weight = {[absolute organ weight (g)/final body weight (g)] × 100}.

**Table 3:** Sperm abnormalities, hormones, and DNA damage in rats intoxicated with LC, TMX, and LC + TMX.

Parameters	Experimental groups			
	Cont	LC	TMX	LC + TMX
Sperm count	86.13 ± 2.92^a^	56.25 ± 3.11^b^	51.11 ± 2.70^b^	34.17 ± 2.33^c^
Sperm motility %	85.16 ± 2.91^a^	65.23 ± 5.51^b^	59.18 ± 3.97^b^	55.19 ± 2.88^b^
Sperm abnormalities %	10.07 ± 0.80^c^	25.13 ± 1.35^b^	27.14 ± 1.06^b^	36.23 ± 1.77^a^
DSP (millions/g testis)	22.69 ± 0.99^a^	15.46 ± 0.30^b^	16.90 ± 0.12^b^	10.01 ± 0.10^c^
Testosterone (ng/mL)	3.51 ± 0.03^a^	1.98 ± 0.02^b^	2.01 ± 0.02^b^	1.02 ± 0.01^c^
LH (ng/mL)	5.32 ± 013^a^	2.43 ± 0.08^b^	2.53 ± 0.06^b^	1.22 ± 0.03^c^
FSH (ng/mL)	16.02 ± 0.11^a^	8.43 ± 0.09^b^	9.10 ± 0.18^b^	5.10 ± 012^c^
Serum 8-OH- 2DG (ng/mL)	1.45 ± 0.10^c^	7.90 ± 0.50^b^	8.02 ± 0.32^b^	10.86 ± 0.63^a^

Values for each treatment group are given as means ± SE; *n* = 7/group. The mean values of LC, TMX, and LC + TMX within a row that did not share common superscript letters (a, b, and c) were substantially different at *P* < 0.05 compared to the control group.

**Table 4:** Thiobarbituric acid reactive-substances (TBARS) and hydrogen peroxide (H_2_O_2_) levels in rats intoxicated with LC, TMX, and LC + TMX.

Parameters	Experimental groups			
	Cont	LC	TMX	LC + TMX
TBARS (nmol/g tissue)	21.75 ± 0.698^c^	28.05 ± 1.133^b^	29.68 ± 0.558^ab^	32.00 ± 1.297^a^
H_2_O_2_ (µmol/g tissue)	62.07 ± 2.26^c^	73.45 ± 2.74^b^	76.11 ± 1.37^b^	85.45 ± 1.93^a^

Values for each treatment group are given as means ± SE; *n* = 7/group. The mean values of LC, TMX, and LC + TMX within a row that did not share common superscript letters (a, b, and c) were substantially different at *P* < 0.05 compared to the control group.

**Table 5:** Semi-quantitative scoring of testicular ultrastructural alterations based on TEM observations.

Parameter	Control	LC	TMX	LC + TMX
Mitochondrial degeneration	−	++	++	+++
Nuclear abnormalities	−	+	++	+++
Cytoplasmic vacuolization	−	++	++	+++
Sertoli–germ cell junction disruption	−	+	++	+++
Basement membrane irregularities	−	+	+	++

## Data Availability

The authors declare that all relevant data that support the findings of this study are incorporated in the manuscript.

## References

[1] Li, L-L. et al. Research advance of time-resolved fluoroimmuno assay for pesticides detection in food. *FMH* **3** (1), 9420105. 10.26599/FMH.2026.9420105 (2026).

[2] Uwamahoro, C. et al. Assessing the risks of pesticide exposure: implications for endocrine disruption and male fertility. *Int. J. Mol. Sci.* **25**, 6945 (2024). 39000054 10.3390/ijms25136945 PMC11241045

[3] Sabzevari, S. & Hofman, J. A worldwide review of currently used pesticides’ monitoring in agricultural soils. *Sci. Total Environ.* **812**, 152344 (2022). 34919921 10.1016/j.scitotenv.2021.152344

[4] Ramanathan, S. et al. Thiamethoxam, a neonicotinoid poisoning causing acute kidney injury via a novel mechanism. *Kidney Int. Rep.* **5**(7), 1111–1113. 10.1016/j.ekir.2020.04.009 (2020). 32647772 10.1016/j.ekir.2020.04.009 PMC7335955

[5] Sakr, S. & Rashad, W. A. Lambda-cyhalothrin‐induced pancreatic toxicity in adult albino rats. *Sci. Rep.* **13**(1), 11562 (2023). 37463968 10.1038/s41598-023-38661-1 PMC10353991

[6] Kandemir, Ö. et al. Carvacrol coadministration ameliorates lambda-cyhalothrin‐induced peripheral neuropathy in rats: Behavioral and molecular evidence. *J. Biochem. Mole Toxicol.*10.1002/jbt.70400 (2025). 10.1002/jbt.70400 PMC12261466 40662443

[7] Lopez-Torres, B. et al. Neurotoxicity induced by the pyrethroid lambda‐cyhalothrin: Alterations in monoaminergic systems and dopaminergic and serotoninergic pathways in the rat brain. *Food Chem. Toxicol.* **169**, 113434 (2022). 36126889 10.1016/j.fct.2022.113434

[8] Zhang, X. et al. Pyrethroids toxicity to male reproductive system and offspring as a function of oxidative stress induction: Rodent studies. *Front. Endocrinol.* **12**, 656106 (2021). 10.3389/fendo.2021.656106 PMC8190395 34122335

[9] Fetoui, H., Garoui, E. M. & Zeghal, N. Lambda-cyhalothrin-induced biochemical and histopathological changes in the liver of rats: Ameliorative effect of ascorbic acid. *Exper Toxicol. Pathol.* **62** (3), 305–312. 10.1016/j.etp.2009.04.004 (2010). 10.1016/j.etp.2008.08.002 18835144

[10] Sood, P. Pesticides usage and its toxic effects: A review. *Indian J. Entomol.* **86**(1), 339–347 (2023).

[11] Qamar, W. et al. Thiamethoxam toxicity: A review in one health perspective. *Kafkas Univ. Vet. Fak Derg*. **29** (5), 557–570. 10.9775/kvfd.2023.30161 (2023).

[12] Yi, L. et al. Evaluation of the risk of human exposure to thiamethoxam by extrapolation from a toxicokinetic experiment in rats and literature data. *Environ. Int.* **173**, 107823 (2023). 10.1016/j.envint.2023.107823 36809708

[13] Abdel-Razik, R. K., Mosallam, E. M. & Hamed, M. A. Deterioration of cytochrome C content and mitochondrial dysfunction in brain of male rats after sub-chronic exposure to thiamethoxam and protective role of N- acetylcysteine. *Alex Sci. Exch. J.* **43** (1), 91–106. 10.21608/asejaiqjsae.2022.223119 (2022).

[14] Abouelghar, G. E. et al. Sublethal toxicity of thiamethoxam insecticide in albino mice: biochemical, oxidative damage and histopathological evaluations. *Adv. J. Toxicol. Curr. Res.* **4** (1), 17–28. 10.37871/ajtcr.id33 (2020).

[15] El-Demerdash, F. M., Jebur, A. B. & Nasr, H. M. Oxidative stress and biochemical perturbations induced by insecticides mixture in rat testes. *J. Environ. Sci. Health Part. B*. **48** (7), 593–599. 10.1080/03601234.2013.774998 (2013). 10.1080/03601234.2013.774998 23581693

[16] El-Demerdash, F. M., Jebur, A. B. & Nasr, H. M. Modulatory effect of *Turnera diffusa* against testicular toxicity induced by fenitrothion and/or hexavalent chromium in rats. *Environ. Toxicol.* **34**, 330–339 (2019). 30578656 10.1002/tox.22688

[17] Jebur, A. B., El-Sayed, R. A., Abdel-Daim, M. M. & El-Demerdash, F. M. *Punica granatum* (Pomegranate) peel extract pre-treatment alleviates fenpropathrin-induced testicular injury via suppression of oxidative stress and inflammation in adult male rats. *Toxics* **11**, 504. 10.3390/toxics11060504 (2023). 37368604 10.3390/toxics11060504 PMC10301163

[18] Shalaby, E. S., Farrag, A. R. H., Saed, E. & SM. G Toxicological potential of thiamethoxam insecticide on Albino Rats and its residues in some organs. *JASMR* **5** (2), 165–172 (2010).

[19] Alsadee, S. A., Mohammed, S. A. & Hasan, A. F. Hepatotoxicity of oral sub-chronic exposure to thiamethoxam and lambda cyhalothrin in rats. *Agricult Rev.* **45**(4), 728–733. 10.18805/ag.RF-350 (2024).

[20] Adamkovicova, M. et al. Sperm motility and morphology changes in rats exposed to cadmium and diazinon. *Reprod. Biol. Endocrinol.* **14**, 1–7 (2016). 27503218 10.1186/s12958-016-0177-6 PMC4977869

[21] Blazak, W. F., Treinen, K. A. & Juniewicz, P. E. Application of testicular sperm head counts in the assessment of male reproductive toxicity. *Method Toxicol.* **3**, 86–94 (1993).

[22] Pfaffl, M. W. A new mathematical model for relative quantification in real-time RT–PCR. *Nucleic Acids Res.* **29**(9), e45 (2001). 11328886 10.1093/nar/29.9.e45 PMC55695

[23] Mohamed, R. A. & Abd El-Rahman, H. A. The effect of an organophosphorus pesticide, chlorpyrifos (from different local sources), on the testicular tissue in adult male albino rats. *EAHT* **40** (1), e2025010. 10.5620/eaht.2025010 (2025). 40400439 10.5620/eaht.2025010 PMC12187380

[24] Ben Abdallah, F., Fetoui, H., Zribi, N., Fakhfakh, F. & Keskes, L. Quercetin attenuates lambda cyhalothrin induced reproductive toxicity in male rats. *Env Toxicol.*10.1002/tox (2011). 10.1002/tox.20762 21887817

[25] Abd-Allah, E., EL-Ghareeb, A. E. W., Hafez, O. & Abd, E. L. Biochemical and histopathological evaluations of thiamethoxam on the male reproductive system. *Egypt. J. Chem.*10.21608/ejchem (2022).

[26] Hassan, A. S., El-Ela, A., Abdel-Aziz, F. I. & A.M Investigating the potential protective effects of natural product quercetin against imidacloprid-induced biochemical toxicity and DNA damage in adults rats. *Toxicol. Rep.* **6**, 727–735 (2019). 31388500 10.1016/j.toxrep.2019.07.007 PMC6676460

[27] Limon-Pacheco, J. & Gonsebatt, M. E. The role of antioxidants and antioxidant related enzymes in protective responses to environmentally induced oxidative stress. *Mut Res.* **674**, 137–147 (2009). 18955158 10.1016/j.mrgentox.2008.09.015

[28] Michelangeli, F., Robson, M. J., East, J. M. & Lee, A. G. The conformation of pyrethroids bound to lipid bilayers. *Biochim. Biophys. Acta*. **1028**, 49–57 (1990). 2207119 10.1016/0005-2736(90)90264-o

[29] World Health Organization. *Cyhalothrin, Environmental Health Criteria, 99* (WHO, 1990).

[30] Al-Omar, M. S. et al. Pyrethroid-induced organ toxicity and anti-oxidant-supplemented amelioration of toxicity and organ damage: The protective roles of ascorbic acid and α-tocopherol. *Int. J. Environ. Res. Public. Health* **17**, 6177. 10.3390/ijerph17176177 (2020). 32854455 10.3390/ijerph17176177 PMC7503327

[31] Hodoșan, C. et al. Pyrethrins and pyrethroids: A comprehensive review of natural occurring compounds and their synthetic derivatives. *Plants* **12**, 4022. 10.3390/plants12234022 (2023). 38068657 10.3390/plants12234022 PMC10707950

[32] Issa, S. Y., Abdel Rahman, S. M., Gaber, Y. M. & Soliman, N. A. H. Toxicological impact of Thiamethoxam on adult male rats: Histopathological, biochemical, and oxidative DNA damage assessment. *Toxicol. Rep.* **14**, 101983 (2025). 10.1016/j.toxrep.2025.101983 40206789 PMC11979398

[33] Mustafa, M. Q. & Jawad, Z. J. M. Evaluating the hepatoprotective potential of ginger ethanolic extract against lambda-cyhalothrin-induced toxicity in male rats. *Iraqi J. Vet. Med.* **48**(2), 26–31 (2024).

[34] Tariba Lovaković, B. et al. Effects of sub-chronic exposure to imidacloprid on reproductive organs of adult male rats: antioxidant state, DNA damage, and levels of essential elements. *Antioxidants* **10**, 1965. 10.3390/antiox10121965 (2021). 34943068 10.3390/antiox10121965 PMC8750738

[35] Yang, H. Y. & Lee, T-H. Antioxidant enzymes as redox-based biomarkers: A brief review. *BMB Rep.* **48**, 200–208 (2015). 25560698 10.5483/BMBRep.2015.48.4.274 PMC4436855

[36] Chandimali, N. et al. Free radicals and their impact on health and antioxidant defenses: a review. *Cell. Death Discov*. **11**, 19. 10.1038/s41420-024-02278-8 (2025). 39856066 10.1038/s41420-024-02278-8 PMC11760946

[37] Sule, R. O., Condon, L. & Gomes, A. V. A common feature of pesticides: oxidative stress—the role of oxidative stress in pesticide-induced toxicity. *Oxid. Med. Cell. Longev. Jan* **19;2022**, 5563759. 10.1155/2022/5563759 (2022). 10.1155/2022/5563759 PMC8791758 35096268

[38] El-Demerdash, F. M. Lambda-cyhalothrin-induced changes in oxidative stress biomarkers in rabbit erythrocytes and alleviation effect of some antioxidants. *Toxicol. Vitro*. **21**, 392–397 (2007). 10.1016/j.tiv.2006.09.019 17137748

[39] Saraivaa, A. S. et al. Assessment of thiamethoxam toxicity to *Chironomus riparius*. *Ecotoxicol. Environ. Saf.* **137**, 240–246 (2017). 27978451 10.1016/j.ecoenv.2016.12.009

[40] Ramchandra, A. M., Chacko, B. & Victor, P. J. Pyrethroid Poisoning. *Indian J. Crit. Care Med.* **23** (4), S267–S271. 10.5005/jp-journals-10071-23304 (2019). 32021002 10.5005/jp-journals-10071-23304 PMC6996658

[41] Ranjbar, M. et al. Gene polymorphisms of epithelial cell-derived alarmins and their effects on protein levels and disease severity in patients with COVID-19. *Genes* **14**, 1721. 10.3390/genes14091721 (2023). 37761861 10.3390/genes14091721 PMC10530834

[42] Habotta, O. A., Ateya, A., Saleh, R. M. & El-Ashry, E. S. Thiamethoxam-induced oxidative stress, lipid peroxidation, and disturbance of steroidogenic genes in male rats: palliative role of *Saussurea lappa* and *Silybum marianum*. *Environ. Toxicol.* **36**(10), 2051–2061. 10.1002/tox.23322 (2021). 34181816 10.1002/tox.23322

[43] Wu, N. et al. Protective benefits and mechanisms of *Phyllanthus emblica* Linn. on aging induced by oxidative stress: a system review. *FMH* **2** (2), 9420029. 10.26599/FMH.2025.9420029 (2025).

[44] Habotta, O., Ateya, A., Saleh, R. M. & El-Ashry, E. S. Thiamethoxam evoked neural oxido-inflammatory stress in male rats through modulation of Nrf2/NF-kB/iNOS signaling and inflammatory cytokines: Neuroprotective effect of Silymarin. *Neurotoxicol* **96**, 28–36 (2023). 10.1016/j.neuro.2023.03.004 36958429

[45] Valavanidis, A., Vlachogianni, T. & Fiotakis, C. 8-hydroxy-2’-deoxyguanosine (8-OHdG): A critical biomarker of oxidative stress and carcinogenesis. *J. Environ. Sci. Health C Environ. Carcinog. Ecotoxicol. Rev.* **27**(2), 120–139 (2009). 19412858 10.1080/10590500902885684

[46] Aitken, R. J. & Roman, S. D. Antioxidant systems and oxidative stress in the testes. *Adv. Exp. Med. Bio*. **636**, 154–171 (2008). 19856167 10.1007/978-0-387-09597-4_9

[47] Turner, T. T. & Lysiak, J. J. Oxidative stress: a common factor in testicular dysfunction. *J. Androl.* **29**(5), 488–498 (2008). 18567643 10.2164/jandrol.108.005132

[48] Lonare, M. et al. Evaluation of ameliorative effect of curcumin on imidacloprid-induced male reproductive toxicity in wistar rats. *Environ. Toxicol.* **31** (10), 1250–1263 (2016). 25758541 10.1002/tox.22132

[49] Ullah, S. et al. Neonicotinoid insecticides inhibit steroidogenesis by disrupting the steroidogenic acute regulatory (StAR) protein expression in mouse Leydig cells. *Environ. Poll.* **244**, 649–656 (2019).

[50] Hamed, I. A. et al. Protective effect of vitamin C against thiamethoxam-induced toxicity in male rats. *Open. Vet. J.* **13** (10), 1334–1345. 10.5455/OVJ.2023.v13.i10.13 (2023). Epub 2023 Oct 31. PMID: 38027408; PMCID: PMC10658022. 38027408 10.5455/OVJ.2023.v13.i10.13 PMC10658022

[51] Li, H. et al. Lambda-cyhalothrin delays pubertal Leydig cell development in rats. *Environ. Pollut*. **242**, 709–717 (2018). 30029170 10.1016/j.envpol.2018.07.033

[52] Xu, X. et al. Oxidative stress and mitochondrial damage in lambda-cyhalothrin toxicity: A comprehensive review of antioxidant mechanisms. *Environ. Pollut.* **338**, 122694 (2023). 37802283 10.1016/j.envpol.2023.122694

[53] Mohamed, A-R., Mohamed, W. A. & Khater, S. I. Imidacloprid induces various toxicological effects related to the expression of 3β-HSD, NR5A1, and OGG1 genes in mature and immature rats. *Environ. Pollut*. **221**, 15–25 (2017). 27914857 10.1016/j.envpol.2016.08.082

[54] Yuan, X. et al. Imidacloprid disrupts the endocrine system by interacting with androgen receptor in male mice. *Sci. Total Environ.* **708**, 135163 (2020). 31780179 10.1016/j.scitotenv.2019.135163

[55] Miller, W. L. Steroidogenesis: unanswered questions. *Trends Endocrinol. Metab.* **28** (11), 771–793 (2017). 29031608 10.1016/j.tem.2017.09.002

[56] Zhang, S. et al. Permethrin may disrupt testosterone biosynthesis via mitochondrial membrane damage of Leydig cells in adult male mice. *Endocrinol* **148**, 3941–3949 (2007). 10.1210/en.2006-1497 17463061

[57] Mosbah, R., Djerrou, Z. & Mantovani, A. Protective effect of *Nigella sativa* oil against acetamiprid induced reproductive toxicity in male rats. *Drug Chem. Toxicol.* **41**(2), 206–212 (2018). 28669218 10.1080/01480545.2017.1337127

[58] Wang, Y. et al. Accumulation and toxicity of thiamethoxam and its metabolite clothianidin to the gonads of Eremiasargus. *Sci. Total Environ.* **667**, 586–593 (2019). 30833257 10.1016/j.scitotenv.2019.02.419

[59] Margret, J. J. & Jain, S. K. The protective role of L-cysteine in the regulation of blood–testis barrier functions: A brief review. *Genes* **15**(9), 1201. 10.3390/genes15091201 (2024). 39336792 10.3390/genes15091201 PMC11430845

[60] Mruk, D. D. & Cheng, C. Y. The mammalian blood–testis barrier: Its biology and regulation. *Endocr. Rev.* **36**(5), 564–591. 10.1210/er.2014-1101 (2015). 26357922 10.1210/er.2014-1101 PMC4591527

[61] Gow, A. et al. CNS myelin and Sertoli cell tight junction strands are absent in Osp/Claudin-11-null mice. *Cell* **99** (6), 649–659. 10.1016/S0092-8674(00)81553-6 (1999). 10612400 10.1016/s0092-8674(00)81553-6

[62] Jiang, B. & Yang, D. Environmental toxins and reproductive health: Unraveling the effects on Sertoli cells and the blood–testis barrier in animals. *Bio Rep.*10.1093/biolreprod/7740689 (2024). 10.1093/biolre/ioae126 39180724

[63] Cheng, C. Y. & Mruk, D. D. The blood–testis barrier and its implications for male contraception. *Pharmacol. Rev.* **64** (1), 16–64. 10.1124/pr.110.002790 (2012). 22039149 10.1124/pr.110.002790 PMC3250082

[64] Bal, R. et al. Effects of the neonicotinoid insecticide thiamethoxam on reproductive system of male rats. *Pest Biochem. Physiol.* **104** (3), 247–253. 10.1016/j.pestbp.2012.09.001 (2012).

[65] Tiwary, R., Richburg, J. H. & Tiwary, R. Mono-(2-ethylhexyl) phthalate reversibly disrupts the blood–testis barrier in pubertal rats. *Toxicol. Sci.* **197** (2), 147–154. 10.1093/toxsci/kfad116 (2024). 37941498 10.1093/toxsci/kfad116 PMC10823777

[66] Abdallah, F. B., Fetoui, H., Zribi, N., Fakhfakh, F. & Keskes, L. Protective role of caffeic acid on lambda cyhalothrin-induced changes in sperm characteristics and testicular oxidative damage in rats. *Toxicology and Industrial Health* **28**, 639 (2012). 22025501 10.1177/0748233711420470

[67] Ahmad, L. et al. Toxico-pathological effects of cypermethrin upon male reproductive system in rabbits. *Pestic. Biochem. Physiol.* **103**, 194–201 (2012).

[68] Vasudha, K., Meghapriya, A. & Kishori, B. Recovery of male reproductive health in cypermethrin-exposed rats by testosterone. *Internat J. Res. Analyt Revie*. **5**, 1–7 (2018).

[69] Hussein, H. K., Elnaggar, M. H. & AlZahrani, N. K. Antioxidant role of folic acid against reproductive toxicity of cyhalothrin in male mice. Glo. *Adv. Res. J. Environ. Sci. Toxicol.* **1** (4), 066–071 (2012).

[70] Bakheet, A. A. et al. Evaluation of nano and conventional forms of lambda cyhalothrin toxicity in rats. In *19th Sci. Cong. Fac. Vet. Med., Assiut Univ., Egypt*, 685–696 (2024).

[71] Agarwal, A. & Prabakaran, S. A. Mechanism, measurement and prevention of oxidative stress in male reproductive physiology. *Indian J. Experim Biol.* **43**, 963–974 (2005). 16315393

[72] Sarry El-Din, M. A., El Ghareeb, A., El-Garawani, I. M., El-Rahman, A. & H. A Induction of apoptosis, oxidative stress, hormonal, and histological alterations in the reproductive system of thiamethoxam-exposed female rats. *Env Sci. Pol. Res.* **30**, 77917–77930 (2023). 10.1007/s11356-023-27743-2 PMC10299933 37266787

[73] Zuscikova, L. et al. Screening of toxic effects of neonicotinoid insecticides with a focus on acetamiprid. *Rev. Toxics* **11**(7), 598. 10.3390/toxics11070598 (2023). 10.3390/toxics11070598 PMC10383352 37505564

[74] Silva, M. H. et al. Cumulative risk assessment of pesticide mixtures: A case study of application to the female reproductive system. *Curr. Protoc.* **2**(4), e400 (2022).35349226

